# HuR Protein in Hepatocellular Carcinoma: Implications in Development, Prognosis and Treatment

**DOI:** 10.3390/biomedicines9020119

**Published:** 2021-01-27

**Authors:** Vasiliki Papatheofani, Georgia Levidou, Panagiotis Sarantis, Evangelos Koustas, Michalis V. Karamouzis, Alexandros Pergaris, Gregorios Kouraklis, Stamatios Theocharis

**Affiliations:** 1 First Department of Pathology, Medical School, National and Kapodistrian University of Athens, 11527 Athens, Greece; vassilikipapatheofani@hotmail.com (V.P.); glevidou@yahoo.gr (G.L.); psarantis@med.uoa.gr (P.S.); alexperg@yahoo.com (A.P.); 2 Second Department of Propedeutic Surgery, Medical School, National and Kapodistrian University of Athens, 11527 Athens, Greece; gkouraklis@med.uoa.gr; 3 Molecular Oncology Unit, Department of Biological Chemistry, Medical School, National and Kapodistrian University of Athens, 11527 Athens, Greece; vang.koustas@gmail.com (E.K.); mkaramouz@med.uoa.gr (M.V.K.)

**Keywords:** HuR, HCC, ELAV, HSC

## Abstract

Hu-antigen R (HuR) is a post-transcriptional regulator that belongs to the embryonic lethal abnormal vision Drosophila-like family (ELAV). HuR regulates the stability, translation, subcellular localization, and degradation of several target mRNAs, which are implicated in carcinogenesis and could affect therapeutic options. HuR protein is consistently highly expressed in hepatocellular carcinoma (HCC) compared to the adjacent normal liver tissue and is involved in the post-transcriptional regulation of various genes implicated in liver malignant transformation. Additionally, HuR protein seems to be a putative prognosticator in HCC, predicting worse survival. This review summarizes the recent evidence regarding the role of HuR in primary liver tumors, as presented in clinical studies, in vitro experiments and in vivo animal models. In conclusion, our review supports the consistent role of HuR protein in the development, prognosis, and treatment of HCC. Additional studies are expected to expand current information and exploit its putative employment as a future candidate for more personalized treatment in these tumors.

## 1. Introduction

Hu-antigen R (HuR) or ELAV (embryonic lethal, abnormal vision Drosophila)-like protein 1 (ELAVL1) is a ubiquitously expressed RNA-binding post-transcriptional regulator, which contains three highly conserved RNA binding domains and belongs to the RNA recognition motif (RRM) superfamily [1,2]; RRM-1 and -2 bind to AU-rich elements (AREs), while RRM3, by binding to the mRNA poly(A) tail, mediates canonical RNA interactions and exists a dimerization interface localized on the α-helical face of RRM3. Significantly, HuR binds to a U-rich sequence, usually located within the 3’ untranslated region (UTR) of the target mRNAs and regulates directly or indirectly their stability, translation, and nucleo-cytoplasmic translocation [3,4].

It is well-established that HuR protein translocates from the cell nucleus (abundantly expressed) to the cytoplasm along with mRNA binding [5], and this translocation is strongly associated with its regulatory function [6]. In addition, a plethora of proteins, microRNAs, hormones [7], cyclic GMP (cGMP)- elevating agents [8], and several drugs appear to alter HuR mRNA and protein levels. Moreover, the ubiquitin-mediated proteasome system’s initiation leads to HuR protein degradation and to caspase-depended mechanisms during apoptotic cell death [9].

Several research studies suggest that alterations in HuR protein levels or its subcellular localization associate with several human diseases, such as pathological inflammation [10], atherosclerosis [11], tissue ischemia [12] and, most significantly, carcinogenesis [13,14]. Moreover, HuR appears to regulate proteins that are involved in several cancer cell functions such as cell cycle regulation, apoptotic cell death, cell signaling, stress response and inflammation [15,16,17].

Many clinical studies have shown that HuR protein levels correlate with a malignant phenotype and/or patients’ prognosis in various cancer types (oral, esophageal, gastric, colorectal, gallbladder, renal, urothelial, pancreatic, lung, breast, cervical, and ovarian cancer). Furthermore, current data suggest the HuR molecule acts as a potential therapeutic target in various carcinomas (glioblastoma, breast cancer), including those of the liver [14,18].

According to the above considerations, the goal of this review is to present the current information regarding the role of HuR protein in hepatocellular carcinoma (HCC), as described by in vitro experiments, in vivo animal models and clinical trials. Initially, an outline of HuR protein expression in liver cell lines and tissues is presented. Afterwards, the unique gene products modulated by HuR protein, either directly (physical interaction among HuR and the target mRNA) or indirectly, are referred. Last, the clinical significance of HuR protein expression and its potential use as a therapeutic intervention against HCC is assessed.

## 2. Hepatocellular Carcinoma (HCC)

HCC is the fifth most frequent cancer worldwide and the third leading cause of cancer death, showing an increased incidence during the last decade [19]. Eight percent (8%) of HCC cases develop in cirrhotic livers and this pre-neoplastic condition represents the most substantial predisposing factor [20]. The molecular pathogenesis of HCC is multifarious [21,22]. The most accepted established hypothesis is a gradual process through which external stimuli provoke genetic modifications in mature hepatocytes, leading to cell death and cellular proliferation/regeneration [19]. Another theory supports that cancer cells are reprogrammed and subjected to metabolic changes, in order to respond to new conditions through post-translational modification (PTM) [23].

## 3. HuR Expression in Normal Liver Tissue and Related Tumor Cell Lines

HuR protein expression has been studied in several human and mouse liver cell lines; mouse liver progenitor 29 cell line MLP29, mouse HCC cell line SAMe-D, human hepatoma cell line HepG2, human HCC cell lines Hep3B, SNU-182, SNU-398, SNU-449, and SNU-475, as well as primary mouse hepatocytes isolated from a male C57BL6 and mainly immortalized normal hepatocytes CRL4020 [24,25]. Embade et al. reported significantly higher HuR protein levels in MLP29 and SAMe-D cell lines than in primary mouse hepatocytes and high HuR expression in the human hepatoma HepG2 cell line [24]. The same applied to the mouse HCC-derived SAMe-D cell line [26] and all human HCC cell lines examined, which showed higher HuR expression levels than normal CRL4020 cells [25]. Furthermore, immunofluorescent analyses of normal versus malignant liver tissue revealed that HuR protein is down-regulated in normal human liver samples and up-regulated in HCC samples of different aetiologies [cirrhotic patients with Hepatitis C (HCV), alcoholic steatohepatitis, and non-alcoholic steatohepatitis (NASH)], and are increased proportionately to their transformation status [27]. Similar observations were reported by Zhu et al. [25]. These findings suggested that HuR protein plays a significant role during malignant hepatocyte transformation and could also represent a novel target for intervention in diverse liver pathologies [27].

## 4. HuR Target Genes and Modulators in Liver Cancer

A summary of mRNA targets of HuR protein is presented in Table 1. Recent information suggests that HuR plays a crucial role in hepatocyte proliferation, differentiation and HCC transformation through post-transcriptional regulation of key transcripts involved in liver function. In particular, HuR protein is associated with the 3′UTR of Methionine adenosyltransferase *MAT2A* mRNA, which along with *MAT1A*, regulates S-adenosylmethionine synthesis (*SAMe*), an essential molecule that is associated with hepatocytes’ proliferation and differentiation [27]. Several studies in HCC highlighted the correlation of *MAT2A* expression with de-differentiation and rapid proliferation of cancer cells. *MAT2A* up-regulation has been associated with liver regeneration during hepatocyte de-differentiation [28,29]. HuR/methyl-HuR and AUF1 have been identified as crucial regulators of hepatic SAMe levels during liver proliferation, de-differentiation and tumor development. This correlation was revealed by studying the expression pattern of specific mRNAs (AU-rich RNA binding factor 1 or AUF1 and HuR) and its association with *MAT1A* and *MAT2A*, mRNA levels. Therefore, HuR/methyl-HuR and AUF1 appeared to control the switch between *MAT1A* and *MAT2A* expression [27].

Furthermore, HuR protein was found to be overexpressed in primary human HCC. Additionally, a significant association between Mdm2 and HuR proteins’ expression in HCC cases related to HCV infection, was noted. Moreover, high HuR protein levels in different cell lines such as SAMe-D and MLP29 were correlated with other cell cycle regulators belonging to HuR targets, such as cyclin A and cyclin D1. Furthermore, knockdown of *Mdm2* in HepG2 and MLP29 cell lines led to a substantial reduction of HuR and cyclin-A protein levels. Also, *HuR* silencing triggered the induction of caspase-3 activation and lowered the number of cells in the S-phase of the cell cycle in several cell lines (MLP29, SAMe-D, HepG2), suggesting that *HuR* ablation promotes apoptosis and cell cycle arrest. Consequently, a cross-talk between Mdm2 and HuR functionality was observed. Interestingly, it has also been demonstrated that HuR was stabilized by Mdm2-depended NEDDylation (NEDDylation is a post-translational modification and it is analogous to ubiquitylation) in at least three lysine amino acid residues, securing its subcellular localization in the nucleus and protection from degradation. Mdm2/NEDD8/HuR axis plays an important role in liver malignant transformation and tumor progression, potentially amenable for cancer therapy [24].

An interesting interaction between HuR protein and LKB1 (serine/threonine-protein kinase 11), a tumor suppressor with recently reported oncogenic functions, has been reported. LKB1 has a crucial role in hepatocyte proliferation and liver regeneration, and its activation in mice lacking the *MAT1A* gene is related to spontaneous development of NASH and HCC. Martinez-Lopez et al. reported that LKB1 phosphorylation regulates the cytoplasmic localization of HuR protein, which in turn, specifically binds to the 3’UTR of HAUSP, therefore stabilizing it and raising its levels [26]. HAUSP is a nuclear ubiquitin-specific protease that targets p53 and Mdm2 proteins as substrates and, in parallel with Mdm2, has a vital role in p53 functionality [31]. The cytoplasmic accumulation of HAUSP allows HAUSP-p53 interaction and leads to a more steady cytoplasmic p53 form, thus, controlling the apoptotic response through this molecule [26].

The interference of HuR protein in the apoptosis of HCC cells was also investigated in another study, which analyzed the interaction between HuR and Fas death receptor [25]. According to this study, HCC cell lines were found to express low FasL levels and were resistant to Fas-FasL stimulation, in contrast to immortalized hepatocytes, which were FasL sensitive. Moreover, it was demonstrated that HuR was interacting with the 3′-UTR of Fas mRNA, blocking its translation. On the contrary, HuR silencing increased the levels of Fas and sensitized HCC cells to FasL. HuR protein seemed to effectively prevent Fas-mediated apoptosis in HCC, suggesting that targeting HuR would stimulate cell apoptosis and reverse tumorigenic properties [25].

Several studies have supported the association between HuR protein levels and cirrhosis, highly increasing the risk of HCC development. Woodhoo et al. suggested that the silence of *HuR* in a cholestatic liver injury model reduced chemoattractant and pro-inflammatory genes’ expression. This led to a reduction of oxidative stress, macrophage infiltration, inflammation, liver damage, and consequently, liver fibrosis. Furthermore, HuR appeared to regulate the activation of hepatic stellate cells (HSC) through PDGF and TGFb [30]. Consequently, the association between HuR protein expression and liver fibrosis is conducted by controlling HSC activation, liver damage, and inflammation.

Other studies also suggested the association between HuR protein and cellular processes such as apoptosis and ferroptosis. Ferroptosis has been recognized as a novel cell death mechanism, characterized by reduced cell size and increased mitochondrial membrane thickness, without the classical apoptotic or necrotic morphology [32]. Increasing protein levels of HuR triggered the initiation of autophagy and autophagosome formation [33], the key mechanism for ELAVL1-depended ferroptosis. Inhibition of autophagy through siRNA against *BECN1* led to ELAVL1-enhanced ferroptosis. The interaction between *BECN1* mRNA and HuR is the primary molecular event, initiating autophagy, increasing autophagic ferritin degradation and consequently leading to ferroptosis. Increasing knowledge on ferroptosis and related molecular mechanisms might lead to putative therapeutic strategies for HSC and liver fibrosis [34].

**Figure 1:**
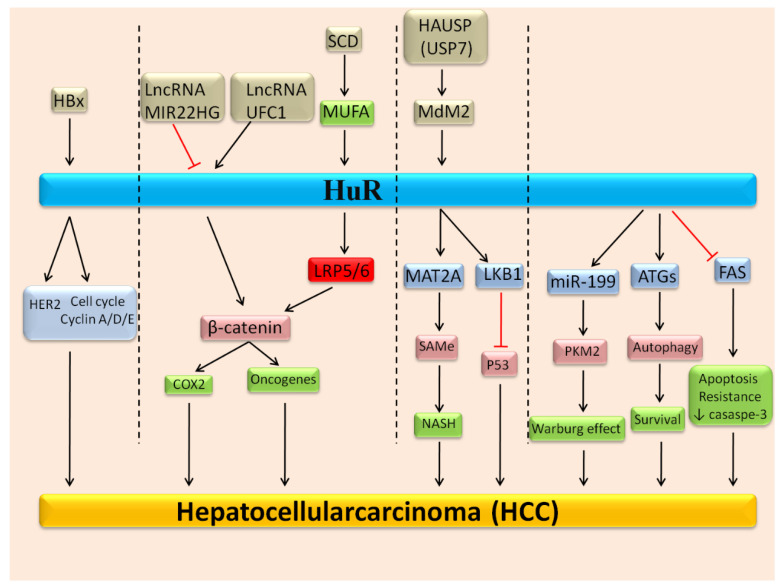
Molecular mechanisms that trigger the activation of HuR and its downstream axis that are participated in the progression of hepatocellularcarcinoma (HCC).

It is well-known that HuR can bind and regulate the mechanisms of subsets of mRNAs such as miRNAs and lncRNAs [35]. Several studies highlighted that HuR mediated the function of *MIR22HG* lncRNA in HCC cells. Recent studies suggested alterations of HuR nucleus/cytoplasm ratio strongly correlated with tumorigenesis [36]. The accumulation of HuR protein within the cytoplasm was associated with negative clinical cancer patients’ outcome [34,37]. In a recent study, Zhang et al. suggested that the *MIR22HG* regulates HuR subcellular localization. Moreover, wild type or mutated *MIR22HG* up-regulation triggered the HuR translocation from the cytoplasm to the cell nucleus. Besides, HuR also appeared to control the *MIR22HG* stability and intervened in the function of *MIR22HG* in HCC. HuR regulated the stabilization of certain tumor suppressors; thus, it may constitute a noticeable target for therapeutic strategies in cancer [38]. HuR protein molecular targets in the liver are represented in Table 1 and Figure 1.

## 5. Clinical Significance of HuR Expression

The clinical significance of HuR expression has been studied in both HCC and other cancers. In HCC, HuR expression levels were significantly associated with advanced clinical stage and were related to low survival rates in patients with early disease stage (I or II) [25]. HuR proved upregulated in breast cancer, and the elevated cytoplasmic HuR expression levels were correlated with high-grade tumors and poor patients’ overall survival (OS) and disease-free survival (DFS) [39]. The elevated cytoplasmic HuR expression levels were also significantly linked to higher tumor stage in colorectal cancer. In gastric cancer, the high nuclear HuR expression levels were correlated with the depth of tumor invasion, TNM stage, and tumor size, while the cytoplasmic HuR subcellular localization was linked to poor patients’ survival [40,41]. Cytoplasmic HuR expression was linked to histological grade, lymph node and distant metastasis in Oral Squamous Cell Carcinoma (OSCC) and lymphatic invasion in thyroid carcinoma [16]. HuR nuclear expression was also associated with reduced DFS in ovarian carcinoma patients. The opposite effect was noted in prostate carcinoma [42]. In lung adenocarcinoma, the elevated cytoplasmic HuR protein expression was linked to poor patients’ clinical outcome [43].

HuR expression levels also influenced chemotherapy resistance. Pancreatic cancer patients with high HuR protein expressing tumors, treated with gemcitabine, showed a significantly longer DFS rate than those with low HuR expression [44]. On the other hand, HuR overexpression in glioma was correlated with drug resistance and increased tumor growth affecting Bcl-2 expression [45]. Additionally, HuR was found to reduce the sensitivity of liver cancer cells to radiotherapy by upregulaing the mRNA expression of mitochondrial transcription factor A (TFAM), which is linked to decreased radiosensitivity [46].

**Figure 2:**
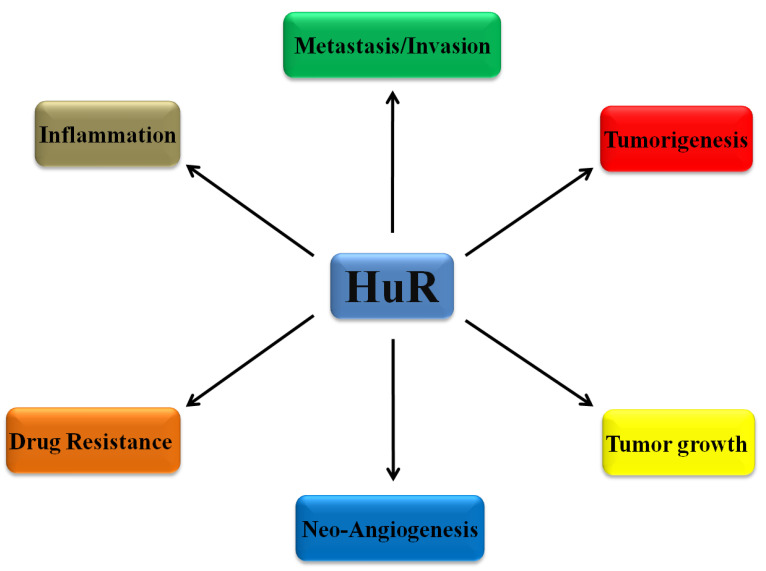
The diverse role of HuR in cancer development and progression through the regulation of the stability and/or translation of target mRNAs that control metastatic invasion properties of cancer cells, angiogenesis, tumor growth, inflammation and tumor resistance against multiple agents.

According to the above mentioned studies, it is prominent that HuR overexpression and cytoplasmic localization is related to worse cancer patients’ prognosis. This is due to the fact that the many stress stimulators, shown to induce HuR shuttling, such as hypoxia [47] and inflammation [48], were found in precancerous and cancerous conditions. The abundantly produced HuR protein binds to mRNAs encoding proto-oncogenes, cytokines, growth factors, and invasion related factors, stabilizing them and eventually establishing a more aggressive cancer phenotype [42]. However, since HuR protein is essential for the differentiation, proliferation, metastasis and survival of HCC cancer cells, the therapeutic employment of HuR targeting might offer us the ability to regulate a wide range of HuR-mediated pro-tumorigenic effects (Figure 2).

## 6. HuR Treatment

A recent article by Liu R et al. summarized different modes of HuR protein function targeting [49]. Targeting HuR protein with small molecules, mainly natural products, have been previously reported, although the exact mechanistic characterization of such compounds and their extensive use in experimental cellular and animal models is still lacking [38,49].

Among such molecules, the most extensively examined MS-444 [50], exerted anticancer effects by affecting HuR protein trafficking and inhibiting its homodimerization and subsequently its ability for RNA binding in different cancer types [51,52,53,54]. Another small molecule with HuR inhibitory activity, KH-3, can inhibit the growth of cancer cells, as well as their metastatic potential in in vitro and in vivo experimental models, by mediating the HuR-FOXQ1 connection [39].

Recently, CMLD-2, a small-agent that directly inhibits HuR protein, seems to display antitumor activity in non-small cell lung cancer (NSCLC) [55,56] and thyroid cancer (TC) [57]. In addition, treatment of NSCLC cell lines (such as A549, HCC827, H1299, H1975) with CMLD-2 triggered G1 phase cell-cycle arrest and apoptosis in a dose-dependent pattern, when compared to its minimal effect on normal fibroblastic cells (MRC-9 and CCD-16 cell lines). CMLD-2 seemed to reduce both *HuR* mRNA and mRNAs for other HuR-dependent proteins in NSCLC cell lines. Furthermore, CMLD-2 treatment reduced the expression levels of several genes, such as *cyclin E*, *Bcl2*, *Bcl*-*XL* and *HuR,* while augmented those of *Bax* and *p27* in NSCLC cells [55,58]. CMLD-2 treatment also activated caspase-9 and -3 and induced PARP cleavage, reduced cell viability and induced apoptosis in TC cell lines (such as BCPAP, K1, 8505 C and/or SW1736), also affecting their migration and colony formation ability. Additionally, CMLD-2 treatment reduced the HuR target protein levels, microtubules-associated protein MAD2, which is upregulated in cancer [57,59]. Similarly, it was also shown that latrunculin A, an actin-depolymerizing macrolide, and a well-known myosin II ATPase inhibitor, blebbistatin, reduced the increased the HuR protein content of HCC cell lines, Huh7 and HepG2, in a time- and dose-dependent pattern. Both inhibitors strongly decreased the abundance of cytoskeletal and membrane-bound HuR protein and conversely triggered its nuclear cell fractions in HCC [60]. In addition, both agents seemed to reduce the expression levels of *COX*-*2*, cyclin A and cyclin D1 mRNAs in HepG2 cells. The decreasing levels of cytoplasmic HuR by blebbistatin or latrunculin-A were correlated with an important sub-cellular localization of HuR mRNA cargo from polysomes to ribonucleoprotein (RNP) particles [61]. In addition, HuR-knockdown seemed to negatively affect the synthesis and migration of prostaglandin E2 in HepG2 cells. Thus, interaction with the actomyosin-dependent HuR sub-cellular trafficking might represent a putative therapeutic option by antagonizing the pathologic posttranscriptional gene expression by HuR protein, and consequently highlighting the beneficial effect of HuR inhibition as chemotherapeutic HCC strategy [60].

Another study investigated the inhibitory effects of N-benzylcantharidinamide, among a number of synthetic analogs of cantharidin agents, on matrix metalloproteinase (MMP)-9-dependent invasive capacity of Hep3B cells. N-benzylcantharidinamide treatment suppressed, dose-dependently, Hep3B cells invasive capacity, due to HuR-mediated reduced MMP-9 mRNA stability [62,63].

Several studies widely used siRNAs against *HuR* in order to investigate its pathophysiological role [14,64]. Recently, Muralidharan et al., developed nanoparticles (NPs) in order to specifically create siRNA, targeting *HuR*. The therapeutic benefits of folate receptor(FR)-α (FRA)-targeted DOTAP:Cholesterol lipid nanoparticles, which carry siRNA against *HuR* (HuR-FNP) was tested in H1299 lung cancer cell line, characterized by high levels of FRA, and in normal lung fibroblast cells (CCD16) with low or negative *FRA* gene expression [65].

In H1299 cells, FNP uptake was significantly higher than in CCD16 cells, implicating a receptor-dose effect. In addition, HuR-FNPs internalization process in H1299 cells was more effective through FRA-mediated endocytosis. HuR-FNP decreased *HuR* mRNA, further augmenting G1 phase cell-cycle arrest and triggering apoptosis in H1299 cells. No similar effect was observed when control siRNA (C-FNP) was applied [65].

Multi-functional NP-based drug delivery systems using folic acid (FA) that trigger simultaneous delivery of several therapeutic agents, such as cisplatin and siRNA for human *HuR* mRNA, were applied in cancer cells with FR-overexpression. The dendrimer-polyethyleneimine-cis-diamminedichloroplatinum-siRNA-folic acid (Den-PEI-CDDP-siRNA-FA) NP machinery was proved beneficial in NSCLC cell lines (A549and H1299), while decreased toxicity to normal lung fibroblast cells (MRC9) [65].

It was documented that a short RNA with AU-rich elements, obtained from C/EBPβ 3’UTR, which bound explicitly to HuR and competed with *C/EBPβ* mRNA, suppressed liver cancer cell growth. Such data support the hypothesis that the suppression of cancer cell growth by 62 nt RNA, which contains the AU-rich elements, might explain the competitive binding to HuR. It was noted that a 62 nt short RNA with 3′UTR derived from *C/EBP beta* mRNA, significantly competed with *C/EBP beta* mRNA itself for the binding of HuR, consituting the reason of C/EBP beta inhibition and consequent suppression of HCC tumor growth [66]. According to this study, short RNA, which specifically inhibits the binding of HuR to its target genes (without affecting other HuR-mRNA binding interactions), might be a putative strategy for HCC and should open novel approaches for the development of anti HCC drugs. The function of the above molecules is summarized in Table 2.

## 7. Conclusions

Increased HuR protein expression is observed in HCCs irrespective of its cause, compared to adjacent normal tissue [25,27].

Current information suggests that in HCC, HuR protein binds to numerous mRNAs encoding proteins implicated in liver function regulation and malignant transformation. Typical examples are the post-transcriptional regulation of MAT2A mRNA, which catalyzes the principal methyl donor’s synthesis and constitutes a crucial controller of hepatocyte proliferation and differentiation SAMe. Another example includes its involvement in the regulatory framework with Mdm2 and NEDD8, inducing growth advantage in HCC cancer cells.

Importantly, HuR protein appears to have clinical importance in HCCs, being correlated with advanced clinical stage. Furthermore, high HuR protein expression is linked to low survival rates of early disease-stage HCC patients [25].

Based on the previously analyzed data, the current study elucidates the significance of HuR protein in the development, prognosis, and treatment of HCC. Recent information on HuR targeting in cell lines of different origin support the notion of future use of small molecules or nanotechnology products in various cancer types, including HCC. Targeting HuR protein, affecting its molecular interactions, represents a unique opportunity to improve patients’ outcome.

In conclusion, further studies are necessary in order to lay the groundwork for setting up a future, prospective and personalized patients’ treatment.

## Figures and Tables

**Table 1:** HuR mRNA targets in liver tissue.

mRNA Target	Function of mRNA Target	Result of HuR Intervention	REF
*MAT2A*	catalyse the synthesis of S-adenosylmethionine (SAMe)	↑ MAT2A promotes hepatocyte de-differentiation in liver regeneration and human HCC	[27]
*Mdm2*	cell cycle regulator	↑ Mdm2 growth advantage of cancer cells HuR is also stabilized by Mdm2-mediated NEDDylation	[24]
cyclin A and cyclin D1	cell cycle regulator	↑cyclin A and D1 growth advantage of cancer cells	[24]
caspase 3	involved in apoptosis	↓ caspase-3 inhibition of apoptosis	[24]
HAUSP	along with Mdm2 modulate p53 function	p53 stabilization regulation of apoptotic response	[26]
*Fas*	involved in apoptosis	↓ Fas inhibition of Fas-mediated apoptosis	[25]
*PDGF* and *TGFb*	hepatic stellate cell activators	promotion of liver fibrosis	[30]

**Table 2:** The different functions of the compounds targeting HuR.

Compound	Function	REF
MS-444	Inhibits the cytoplasmic translocation of HuR,	[50,53]
N-Benzylcantharidinamide	Inhibits the cytoplasmic translocation of HuR,	[62]
Latrunculin A	Inhibits the cytoplasmic translocation of HuR,	[60,61]
Blebbistatin	Inhibits the cytoplasmic translocation of HuR,	[60,61]
HuR-FNP	Targets HuR’s mRNA and regulates its expression.	[65]
CMLD2	Inhibits HuR binding to target mRNAs	[57]
KH3	Inhibits HuR binding to target mRNAs	[39]
Short RNA with AU-rich elements, obtained from C/EBPβ 3′UTR	Inhibits HuR binding to target mRNAs	[66]

## Data Availability

No new data were created or analyzed in this study. Data sharing is not applicable to this article.
